# The use of rubber dam in the survival of RMGIC restorations in primary molars: a 30-month randomized controlled clinical trial

**DOI:** 10.1590/1807-3107bor-2024.vol38.0009

**Published:** 2024-01-05

**Authors:** Vanessa dos Santos BRUM, Maria Luiza Vieira BORGES, Nicole Marchioro dos SANTOS, Camila KAUFMANN, Jonas de Almeida RODRIGUES

**Affiliations:** (a) Universidade Federal do Rio Grande do Sul – UFRGS, School of Dentistry, Department of Surgery and Orthopedics, School of Dentistry, Porto Alegre, RS, Brazil.

**Keywords:** Dental Caries, Rubber Dams, Survival Rate, Glass Ionomer Cements, Tooth, Deciduous

## Abstract

This study was a randomized controlled clinical trial with two parallel arms and the objective was to compare the survival of resin modified glass ionomer (RMGIC) restorations in primary teeth using rubber dam or cotton roll isolation after a 30-month follow-up period. Ninety-two children (mean age 6.8 ± 1.37) and 200 primary molars with occlusal or occluso-proximal cavitated dentin caries lesions were randomly assigned into two groups: cotton rolls and rubber dam. All lesions were restored using RMGIC (RIVA Light Cure) after selective caries removal. Restorative failure and lesion arrestment were evaluated by two independent, trained, and calibrated examiners through clinical and radiographic examinations. The Kaplan-Meier test was used to assess the survival of restorations and Cox regression was used to assess the association of risk factors with restorative failure. There was no significant difference in survival rates between groups (p = 0.17). Older age (HR = 2.81 [95%CI: 1.47–5.44]) and higher rate of gingival bleeding (HR = 0.47 [95%CI: 0.23–0.99]) were associated with restorative failure. No patient had painful symptoms, pulp outcomes, or radiographic changes compatible with lesion progression. The use of rubber dam isolation did not increase the survival rate of occlusal and occluso-proximal restorations using RMGIC in primary molars after 30 months of follow-up. Since the survival is not influenced by the type of isolation, the professional can safely choose the appropriate technique for each case, considering his experience and preferences, as well as those of the patient.

## Introduction

Dental caries is a multifactorial and dynamic disease and remains one of the most prevalent chronic diseases in children, with the primary molars being the most affected in this group.^1,2^ In restorative treatment, the aim of carious tissue removal is to create conditions for restoration by preserving nondemineralized and remineralizable tissue. In this sense, nonselective or complete caries removal is considered overtreatment and is no longer indicated, with selective removal being the currently recommended approach for dentin lesions.^3^

Composite resin and glass ionomer cement are the most frequently indicated restorative materials for primary teeth. Selection should be guided by factors such as the location and extent of the lesion, the individual caries risk, the patient’s condition, moisture conditions, the professional’s ability, and professional and patient preferences.^3^ In a recent systematic review that evaluated the longevity of restorations with different materials after selective caries removal in posterior primary teeth, no significant difference was observed between composite resin and resin-modified glass ionomer cement (RMGIC), which, on the other hand, showed greater survival rates than conventional chemically activated glass ionomer cement.^4^ Therefore, the particularities of the restorative material must also be considered when selecting the isolation technique. The composite resin restorative technique recommends the use of rubber dam isolation to reduce bacterial contamination and control humidity in the operative field.^5^ For glass ionomer, isolation is recommended, but the manufacturer does not specify whether the isolation should be absolute or relative.

Absolute and relative isolation techniques have advantages and disadvantages, with both used to isolate the operative field from moisture. To properly perform absolute/rubber dam isolation, a rubber dam, clamp, and rubber dam frame are necessary, and anesthesia is often used to reduce discomfort.^6^ When performed by an experienced dentist, rubber dam isolation causes less stress in children and adolescents, protects against aspiration, improves the working field, and protects soft tissue.^7^ On the other hand, relative isolation with cotton rolls requires less operative time, causes less discomfort to the patient, and reduces cost, requiring only cotton rolls and a saliva ejector. A clinical trial concluded that the use of rubber dam did not increase the success of Class II ART restorations with glass ionomer cement in primary teeth, and suggested that pediatric dentists can continue to perform restorations in occluso-proximal cavities using cotton rolls without affecting their survival rates.^8^ In another study, a higher survival rate of restorations was observed with the rubber dam method compared to cotton roll isolation.^9^ A systematic review indicated a higher survival rate at 6 months for restorations of non-carious cervical lesions performed with rubber dam isolation. In primary molars, the use of a rubber dam led to a lower risk of failure at two years for proximal atraumatic restorative treatment, but with very low quality evidence.^10^ This review was recently updated and suggests that the use of a rubber dam could lead to a lower failure rate of direct restorations (composite resin restoration of non-carious cervical lesions and proximal atraumatic restorations) compared to the use of cotton rolls,^11^

The present randomized controlled clinical trial aimed to compare the survival of occlusal and occluso-proximal RMGIC restorations in primary teeth using rubber dam or cotton roll isolation after a 30-month follow-up period. The null hypothesis was that rubber dam isolation does not increase the survival rate of restorations in primary molars. In addition, plaque index and gingival bleeding, caries activity, and risk factors associated with restorative failure were also evaluated.

## Methodology

### Ethical considerations

This study was approved by the Local Research Ethics Committee (CAAE 80465617.6.0000.5347), conducted in accordance with the 1964 Helsinki Declaration, and registered in the ReBEC Platform (RBR-8hcg2c - Does the method of tooth isolation influence the longevity of restorations?). After explanation of the study, all participants and their parents or legal guardians signed a written informed consent.

### Trial design and participants

This was a single-blind randomized controlled clinical trial with two parallel arms and its report followed the guideline proposed in the CONSORT 2010 Statement.^12^

The sample size calculation was based on a previous study^8^ performed on primary molars that evaluated the survival of occluso-proximal atraumatic restorations under rubber dam and cotton roll isolation. Considering a superiority design, a sample of 99 teeth was defined per group (a total of 198 teeth), using a test power of 80%, significance level of 5%, a success rate of 61.9% in the cotton rolls group (deemed as control group). A 30% sample loss and 20% cluster effect were considered. The software used was IBM SPSS 20.0. Survival rates are expected to be the same regardless of the type of isolation.

Between December 2018 and May 2019, 197 children treated at the University Children’s Clinic were clinically and radiographically evaluated, totaling 1576 primary teeth. Two examiners (CSS and NMS), trained and calibrated according to ICDAS scores^13^and for caries activity according to visual-tactile criteria,^14^ performed clinical examinations and standardized modified bitewing radiographs using an Emmenix Film Holder (Hager & Werken, Duisburg, North Rhine-Westphalia, Germany), which also allows periapical evaluation through film displacement. The examiners were trained to perform the clinical examination through an expository class with photographs. Furthermore, 20% of the sample was examined before the beginning of the study and again after 2 weeks by the same examiners and the results were compared. The inter-examiner Kappa value was 0.80 and intra-examiner was 0.69 (CSS) and 0.83 (NMS).

Children who presented at least one cavitated dentin caries lesion on the occlusal or occluso-proximal surface of vital teeth and without signs and symptoms of irreversible pulp changes were included. Radiographically, included teeth presented at least two-thirds of the root visible and the lesion depth was in the outer or middle third of the dentin^15^. Teeth that presented spontaneous pain, fistula, mobility not compatible with the period of root resorption, and advanced rhizolysis (resorption > 2/3), as well as patients with systemic conditions were not included. During the study, patients who moved from the city or who no longer wanted to participate were excluded. At the end of this process, 92 children met the inclusion criteria and were included in the study, totaling 200 primary teeth.

### Randomization and blinding

A simple randomization was performed. The individual tooth was the randomization unit, and a numerical sequence for each was generated on the website randomization.com. In cases where the same patient had more than one tooth included in the study, each tooth was treated in a different appointment. The numerical sequence, with the indication of the treatment, were placed in sequentially numbered opaque sealed envelopes. A third person, not directly involved in the study, accessed the envelope with the information about the treatment to be performed and communicated it to the operator only when the patient was already seated in the chair, who then performed the randomly selected isolation technique followed by the restorative treatment.

### Interventions

Prior to treatment, all patients received professional prophylaxis and oral hygiene instruction with toothbrush, dental floss, and fluoridated toothpaste (1100 ppm F), as well as dietary counseling. Teeth were then isolated and occlusal and occluso-proximal lesions were restored by two pediatric dentists (CSS and NMS) following a protocol and according to randomization, as follows:

*Rubber dam group (RD)*: The participants received topical anesthesia followed by local infiltrative anesthetic technique, and the tooth to be restored was isolated with rubber dam, dental clamp, and Ostby arch. For occluso-proximal lesions, in addition, a Tofflemire matrix band number 1 with a universal Tofflemire matrix retainer was used (Tofflemire, New York, USA). The selective caries removal to soft or firm dentin^3^ was performed using a sharp hand excavator and round steel bur, reaching hard enamel and dentin on the periphery and leaving soft carious dentin on the pulpal surface of the cavity, according to the clinical hardness criteria and radiographic depth. The cavity was previously conditioned and then restored with RMGIC (RIVA Light Cure - SDI, Victoria, Australia) according to the manufacturer’s instructions using an appropriate spatula to insert the material. For cavities deeper than 1.8 mm, the material was applied in two layers. A light-curing device (Emitter C - power 1250 mW/cm^2^, Schuster, Brazil) was used to light-cure the material for 20 seconds per layer. At the end, finishing and polishing was performed with diamond drills and silicone tips.

*Cotton rolls group (CR):* The tooth to be restored was relatively isolated with cotton rolls and a saliva ejector, and the restorative technique was performed exactly as described for group RD. In case of deep caries, when participants reported pain, they were submitted to topical anesthesia followed by an infiltrative local anesthesia.

### Follow-up and radiographic analysis

To assess the survival of restorations, patients were clinically and radiographically evaluated after approximately 6, 9, 12, 18, 24, and 30 months.

Restorative material integrity was the main outcome variable. Two trained, calibrated pediatric dentists, blind for the treatment, performed the longitudinal evaluation according to the USPHS criteria^16,17^ (Table 1). The kappa inter-examiner value was 0.75 and the intra-examiner values were 0.89 (JT) and 0.92 (SW). Restorative failure was identified when criteria I and VII obtained a score C and criterion IX obtained a scored B. In these cases, the teeth were submitted to treatment as indicated (restoration, endodontic treatment, or extraction) and failure was recorded. In cases when criterion III was classified as a C score, the restoration was performed again according to the group in which the tooth was initially allocated, and evaluation continued.

**Table 1:** Summary of criteria (USPHS).^16^,^17^

Criteria	Test procedure	USPHS description	Score
I. Retention	Visual inspection with mirror at 18 inches	Complete retention of restoration	Alpha (A)
		Mobilization of the restoration, still present	Bravo (B)
		Loss of the restoration	Charlie (C)
III. Marginal integrity	Visual inspection with mirror at 18 inches	Absence of discrepancy at probing	Alpha (A)
		Presence of discrepancy at probing, without dentin exposure	Bravo (B)
		Probe penetrates in the discrepancy at probing, with dentin exposure	Charlie (C)
VII. Postoperative sensitivity	Ask patients	Absence of dentinal hypersensitivity	Alpha (A)
		Presence of mild and transient hypersensitivity	Bravo (B)
		Presence of strong and intolerable hypersensitivity	Charlie (C)
IX. Secondary caries	Visual inspection with explorer and mirror, if needed	No evidence of caries	Alpha (A)
		Evidence of caries along the marginal of the restoration	Bravo (B)

In all return visits, visible plaque index (VPI) and gingival bleeding index (GBI)^18^ were recorded, and after professional prophylaxis, dental caries was assessed according to ICDAS scores and caries activity according to visual-tactile criteria. Lesions were also evaluated radiographically by standardized modified bitewing radiographs and visually classified by a senior researcher (also blind to treatment groups) as “progressed” or “arrested”, considering increased or stable radiolucency observed in darkroom conditions on the negatoscope. Teeth that showed radiographic progression of the caries lesion were also submitted to appropriate treatment (restoration, endodontic, or extraction) and failure was recorded.

No important changes were made to methods were made after the study began.

### Statistical analysis

Multivariate analysis was performed using Cox regression to assess the association of risk factors with restoration failure. Age and Decayed, Missing, and Filled Teeth (dmft) were dichotomized by the median, and the cutoff point established for VPI and GBI was 10% in accordance with Trombelli et al^18^ Variables with p-values less than or equal 0.20 in the univariate model were included in the multivariate analysis. The Kaplan-Meier test was used to evaluated the differences in survival rates of restorations between groups. A paired t-test was performed to analyze intra-group VPI and GBI. All analyses were performed considering a significance level of 5% and with an appropriate statistical software (IBM SPSS 20.0). Sample power was calculated by a blinded researcher (JAR) using the ‘powerMediation’ package from software R, version 3.5.1 (R Foundation for Statistical Computing, Vienna, Austria)

## RESULTS

Ninety-two children were included in the study (mean age 6.8 ± 1.37); 39 boys (42.4%) and 53 girls (57.6%). In total, 200 cavitated lesions were treated (100 per group). The power of the test was 80.23%

Table 2 describes the characteristics of the sample.

**Table 2:** Sample characteristics according to treatment groups at baseline.

Variable	Cotton rolls	Rubber dam	p-value*
Gender			
Male	40	43	0.66
Female	60	57	
VPI			
< 10%	9	13	0.36
≥ 10%	91	87	
GBI			
< 10%	40	38	0.77
≥ 10%	60	62	
Dmf-t			
< 4	17	20	0.58
≥ 4	83	80	
Age (years)			
< 6.6	45	50	0.47
> 6.7	55	50	
Restored surfaces			
1 surface	60	59	0.88
2 surfaces	40	41	
Arch			
Upper	42	55	0.06
Lower	58	45	
Side			
Right	53	50	0.67
Left	47	50	
Molar			
1^st^ molar	43	44	0.88
2^nd^ molar	57	56	

*Chi-square test; VPI: visible plaque index; GBI: gingival bleeding index. dmft: Decayed, missing, and filled teeth

**Figure 1:**
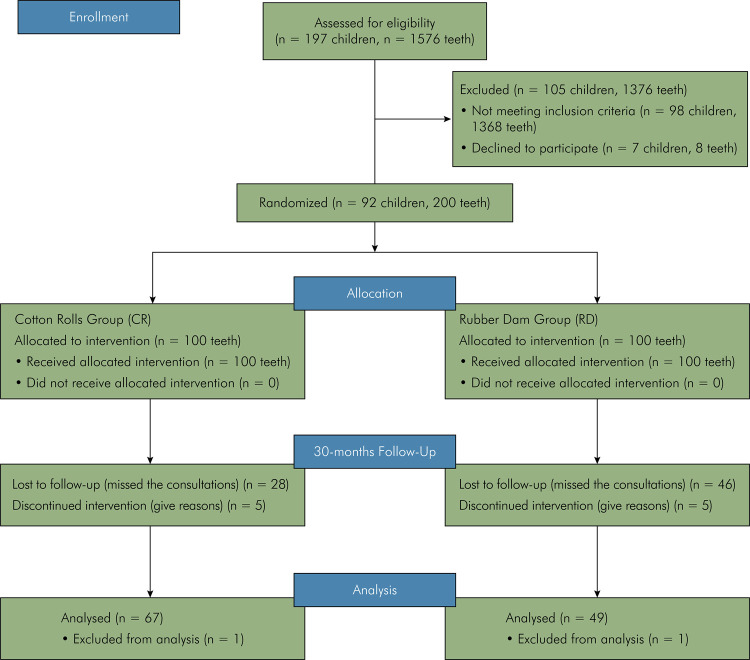
Consort Flow Diagram

After the 30-month follow-up period, 116 restorations were evaluated (49 from group RD and 67 from group CR) in 55 patients (a loss of 42%). The number of patients, teeth allocated to each group, and sample loss in the period are shown in the flowchart (Figure 1).

**Figure 2:**
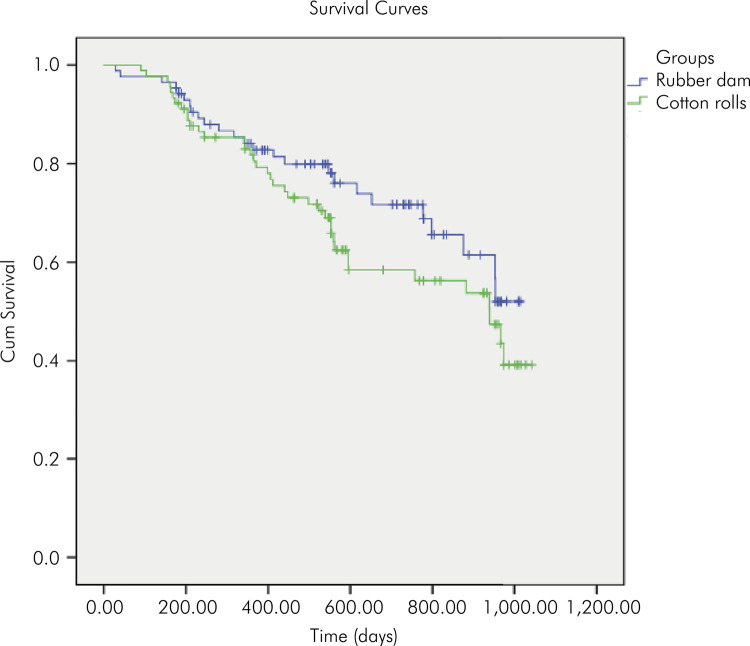
Kaplan-Meier survival curves

The overall success rate was 45.69% (48.98% for the rubber dam group and 43.28% for the cotton rolls group). The Kaplan-Meier survival curve is presented in Figure 2. Mean estimated survival time was 26.7 months for RD [95%CI: 24.3–29] and 24.7 months for CR [95%CI: 22.2–27.2]. The log-rank test was not significant (p = 0.17). No lesions progressed radiographically.

According to USPHS criteria, at 30 months, there were a total of 16 failures in group CR (24.2%) and 17 in group RD (35.4%) as per Criterion I (retention), 10 in group CR (15.1%) and 7 in group RD (14.6%) according to Criterion III (marginal integrity), and 10 in group CR (15.1%) and 3 in group RD (6.2%) according to Criterion IX (caries lesion adjacent to restoration; secondary caries). Postoperative sensitivity (Criterion VII) was not reported by any of the children in either group.

Cox regression (Table 3) shows the association of risk factors (treatment; age; sex; dmft; teeth; lesion location; lesion depth, lesion extension; VPI and GBI) with restoration failure. Older age and GBI were positively associated with restoration failure in both groups, indicated in the table by the asterisk.

**Table 3:** Cox univariatea and multivariateb regression.

Risk factors	Hazard Ratio	p-value	Hazard Ratio	p-value*
	
	(95%CI)^a^		(95%CI)^b^	
Gender				
Male	1.00	0.72	-	-
Female	0.89			
	(0.49–1.63)			
Age (years)				
< 6.6	1.00	0.01*	1.00	0.00*
> 6.7	2.04		2.81	
	(1.13–3.67)		(1.47–5.44)	
Treatment				
Rubber dam group	1.00	0.34	-	-
Cottom rolls group	0.76			
	(0.43–1.33)			
Restored surfaces				
1 surface	1.00	0.89	-	-
2 surfaces	1.04			
	(0.57–1.88)			
Arch				
Upper	1.00	0.86	-	-
Lower	0.95			
	(0.55–1.65)			
Side				
Right	1.00	0.29	-	-
Left	0.74			
	(0.42–1.29)			
Molar				
1^st^ molar	1.00	0.20*	1.00	0.16
2^nd^ molar	0.70		0.64	
	(0.40–1.22)		(0.34–1.19)	
VPI				
< 10%	1.00	0.38	-	-
> 11%	0.76			
	(0.42–1.38)			
GBI				
< 10%	1.00	0.14*	1.00	0.04*
> 11%	0.61		0.47	
	(0.32–1.17)		(0.23–0.99)	
DMFT				
< 3	1.00	0.98	-	-
> 4	0.61			
	(0.34–1.09)			

^a^ univariate; ^b^ multivariate; VPI: visible plaque index; GBI: gingival bleeding index; dmft: Decayed, missing, and filled teeth; *paired t-test.

To analyze the variation in VPI, GBI, and dmft, the paired t-test was used, which indicated a significant improvement in VPI from baseline to the 30-month follow-up (Table 4). As for the GBI and dmft, we observed a significant worsening after 30 months of follow-up.

**Table 4:** Variation in sample and group VPI, GBI, and dmft.

Variable	Paired differences					p-value*

	Mean	SD	Std. Error Mean	95%CI off the difference		

				Lower	Upper	
VPI						
VPI initial – 30 m	0.06	0.23	0.02	0.02	0.11	0.003**
GBI						
GBI initial – 30 m	-0.08	0.23	0.02	-0.12	-0.04	0.000**
Dmf-t						
Dmf-t initial – 30 m	-4.04	2.17	0.20	-4.44	-3.64	0.000**
Cotton rolls						
VPI initial – 30 m	0.06	0.24	0.03	0.00	0.12	0.026**
GBI initial – 30 m	-0.09	0.22	0.02	-0.15	-0.39	0.001**
Dmf-t initial – 30 m	0.46	2.28	0.27	-0.09	1.02	0.102
Rubber dam						
VPI initial – 30 m	0.06	0.23	0.03	-0.00	0.12	0.072
GBI initial – 30 m	-0.07	0.25	0.03	-0.14	-0.00	0.047**
Dmf-t initial – 30 m	0.95	2.36	0.33	0.28	1.63	0.007**

VPI: visible plaque index; GBI: gingival bleeding index. dmft: decayed, missing, and filled teeth.

*Paired t-test; **Difference among the groups is expressed as p < 0.05.

## Discussion

This is the first randomized controlled clinical study that evaluated the survival of RMGIC restorations over a long period comparing two isolation techniques. A recently updated systematic review concluded, with low-certainty evidence, that rubber dam isolation may lead to a lower failure rate of restorations compared to cotton roll. All included studies were at high risk of bias, therefore, further randomized controlled trials with longer follow-up periods were suggested to make a robust conclusion about the effect of isolation type in different restorative treatments.^11^

In 2021, the 9-month interim evaluation of the present randomized clinical trial found no significant difference between the techniques, as rubber dam isolation did not increase restoration survival rates and was not associated with arrestment of cavitated carious lesions in dentin.^20^ After 30 months of follow-up, the results still demonstrate that the use of rubber dam isolation did not improve the survival rate of restorations performed with RMGIC in primary molars. Therefore, the results support the null hypothesis of this study. In both follow-up periods, we did not find radiographic signs of progression of carious lesions in either groups.

The results of the present study are in agreement with Carvalho et al.,^8^ who performed proximal restorations in primary molars using the atraumatic restorative treatment (ART) technique with either rubber dam or cotton roll isolation. The authors also did not observe a significant difference between the two isolation methods and suggested that saliva contamination is not the main cause for occluso-proximal ART restoration failures. The use of rubber dam isolation to perform proximal restorations in primary molars did not affect their survival, and can be seen as a factor that compromises the atraumatic aspect of the proposed technique, as it causes more discomfort for patients. Furthermore, the amount of infected dentin removed from the cavity using the ART technique and the manipulation of restorative materials can influence the success rate of the restoration. Thus, the survival rate of restorations with GIC does not seem to be influenced by the isolation technique used during operation but is more likely associated with other factors inherent to the other steps of the restorative procedure.

Kemoli et al.^9^ evaluated the influence of cotton roll and rubber dam isolation on the survival of proximal restorations under the ART technique in primary molars using three different types of glass ionomer cements (Fuji IX, Ketac Molar Easy mix, and Ketac Molar Aplicap). In contrast, after two years of follow-up, the authors concluded that the survival rate of restorations performed under rubber dam isolation was higher than those performed under cotton rolls isolation, irrespective of the type of GIC material.

In another study from Brazil, cotton roll isolation was shown to be non-inferior when compared to rubber dam for longevity of composite resin restorations in primary molars after two years.^21^ Despite the different restorative material (Bulk fill composite resin), the findings are in agreement with the present study, with similar sample size and follow-up period. In addition, Olegario et al.^21^ points out that the use of rubber dam has disadvantages such as higher cost and longer procedure time.

High sugar intake and poor oral hygiene are common behaviors in pediatric patients at high risk of caries, which contribute to the development of the disease. The factors that cause primary caries lesions are the same that lead to the development of lesions adjacent to the restorations. Collaboration between the dentist, patient, and family are required to modify such behaviors that lead to early failure of restorative treatments.^22^ Chisini et al.^23^ indicated that the presence of carious lesions adjacent to the restorations is the main factor responsible for the failure of treatments with composite or glass ionomer materials, suggesting that the release of fluoride by the GIC did not affect the longevity of restorations. In the present study, ten failures were found in the CR group according to criterion IX (secondary caries) and ten according to criterion III (marginal integrity).

The Cox regression analysis showed that there was an association of restorative failure with GBI and older age. Such age-related findings can be explained by the age of the patients at the time of dichotomization (7 years), as with growth and greater manual skills, children begin brushing their own teeth instead of their parents, worsening hygiene.

In nine months of follow-up, we observed an improvement in the GBI of patients participating in this randomized controlled clinical trial^20^, which can probably be explained by the treatment of all dental problems and the training of children and their families on adequate oral hygiene at study enrollment. However, after 30 months of follow-up, the worsening in dmft and GBI may be related to the fact that improvement of the indexes requires motivating the patient to consistently perform adequate oral hygiene.^24^

The difficulty in returning patients for follow-up and the COVID-19 pandemic, including the temporary suspension of clinical dental care at the university and people avoiding the service, resulted in an unexpectedly high sample loss. We consider that these were the main limitations found during the evaluations. Therefore, a health promotion and empowerment approach to performing adequate oral hygiene is necessary, as patients on leave for extended periods may have difficulty maintaining adequate oral hygiene, worsening gingival bleeding and consequently negatively affects the longevity of restorations.

Throughout the entire follow-up period, there was no radiographic progression of carious lesions. Therefore, restoration failure does not always imply lesion progression. In cases that required reintervention, the restorative material remained in the cavity, and the restorative material needed repair and not replacement,^25^ in line with a minimal intervention approach. In addition, reviews point to a difficulty in assessing the restoration survival due to differences in the criteria used.^4,10^ In the present study, the USPHS criterion was used to assess survival rates, which allows important failures in RMGIC restorations to be recorded by evaluating aspects such as retention, marginal integrity, secondary caries, and postoperative pain. This criterion was also used in other previous studies.^17,20,25^

In Pediatric Dentistry, we must take into account that factors related to the patient and the management of their behavior may also compromise dental care and the performance of therapeutic techniques.^26^ Especially in children, the time required to perform procedures is an important factor, and the psychological impact and discomfort generated by more invasive treatments must also be considered.^27^ Therefore, the results of the present study emphasize the possibility and advantages of using cotton roll isolation to restore dentin cavities with RMGIC.

The survival of restorations can vary according to the different factors that affect the technique. The use of rubber dam isolation is associated with reducing stress in children and adolescents, in addition to reducing the time needed to carry out the treatment. However, once it is clear that rubber dam isolation does not increase survival rates, it is also important to consider the professional’s experience, as well as their treatment preferences, which may directly affect the execution of the isolation technique and the results obtained^7,28^. In addition, future studies comparing isolation techniques using other materials such as conventional, bulk, or flow resins may be important.

## Conclusion

The use of rubber dam isolation did not improve the survival rate of occlusal and occluso-proximal restorations performed with resin-modified glass-ionomer in primary molars after 30 months of follow-up. Since the survival is not influenced by the type of isolation, the professional can select the appropriate technique for each case or patient according to their preference and experience as well as those of the patient. Restorative failure was associated with worsening gingival bleeding rates; therefore, a health promotion approach and hygiene instruction should be strongly encouraged.
